# Predisposing Factors and Acute Precipitants for Race/Ethnicity-Related Resident-to-Resident Aggression

**DOI:** 10.1093/geroni/igaf122.873

**Published:** 2025-12-31

**Authors:** E-Shien Chang, Hannah Mason, Sara Czaja, Karl Pillemer, Mark Lachs, Tony Rosen

**Affiliations:** Weill Cornell Medicine, New York, New York, United States; Weill Cornell Medicine, New York, New York, United States; Weill Cornell Medicine, New York, New York, United States; Cornell University, Ithaca, New York, United States; Weill Cornell Medicine, New York, New York, United States; Weill Cornell Medicine / New York-Presbyterian Hospital, New York, New York, United States

## Abstract

Resident-to-resident aggression (RRA) is a prevalent form of mistreatment in long-term care facilities. Some episodes of RRA are motivated by racial or ethnic differences between residents, but little research has investigated this aspect of the phenomenon. Guided by a theory-informed perspective, we qualitatively explored the predisposing factors and acute precipitants of this race/ethnicity-related resident-to-resident aggression (RE-RRA), defined as RRA motivated by racial or ethnic differences or conflicts that would not have occurred between residents of the same race or ethnicity. We conducted focus groups (n = 9) and in-depth interviews (n = 12) with 81 clinical and non-clinical staff members at two nursing homes in New York. Qualitative data was coded using a thematic approach. We identified nine significant types of RE-RRA, ranging from physical violence and microaggression to racial avoidance. Consistent with previous research, we found 20 predisposing factors and 14 acute precipitants contributing to RRA overall that contributed to RE-RRA. Notably, 10 predisposing factors and 8 precipitants were uniquely drivers of RE-RRA. We synthesized these findings into a conceptual framework to illustrate how distinct factors operate across resident, staff, facility, and structural levels to cause RE-RRA. Our findings provide the preliminary foundation for developing necessary prevention and intervention programs and emphasize the need to consider distinct race/ethnicity-related dynamics in RRA prevention and intervention research. Also, this represents a critical area of focus to enhance the quality of care among racially and ethnically diverse long-term care residents.

